# Micro- and nanoscale effects in biological and bioinspired materials and surfaces

**DOI:** 10.3762/bjnano.17.14

**Published:** 2026-01-28

**Authors:** Thies H Büscher, Rhainer Guillermo Ferreira, Manuela Rebora, Stanislav N Gorb

**Affiliations:** 1 Department of Functional Morphology and Biomechanics, Kiel University, Am Botanischen Garten 1–9, 24098 Kiel, Germany; 2 Lestes Lab, Federal University of Triângulo Mineiro, Uberaba, Minas Gerais, Brazil; 3 Dipartimento di Chimica, Biologia e Biotecnologie, University of Perugia, Via Elce di Sotto 8, 06121 Perugia, Italy

**Keywords:** adhesion, bioengineering, functional morphology, material properties, medical coatings, microstructures, nanostructures, optics, structure–function relationships

Micro- and nanostructures play a crucial role in shaping the physical and functional properties of living organisms. They are the basis of key biological phenomena, such as coloration, adhesion, and mechanical stability, and influence the way how organisms interact with light, fluids, and forces at different scales. The surfaces of any living organism constantly engage with the environment and face a wide range of challenges. To meet these challenges, the surfaces of any organism must often simultaneously fulfil multiple functions and adapt to various environmental pressures, which involve complex interactions between surface structures and their surroundings at nano-, micro-, and macroscales [1]. The physical constraints shaping such interactions are complex for all organisms, including bacteria, plants, and animals and of relevance across all habitats [2]. Understanding these interactions and the functionality of hierarchical structures aids in understanding the principles of biological design and inspires advances in biomimetics, mechanical engineering, and materials science.

Biomimetics seeks to obtain knowledge on how these structural and material property adaptations affect surface performance and to draw inspiration from biological solutions for technology [3]. By studying multiscale structures and mechanisms in biological systems, biomimetics enables the translation of the fundamental principles into technological solutions for practical uses. In this context, bioinspired nanotechnology plays a vital role in generalizing nanoscale properties and processes in biology to engineer functional surfaces and interfaces across different scales.

In May 2023, the Beilstein Nanotechnology Symposium titled “Functional Micro- and Nanostructured Surfaces: from Biology to Biomimetics” brought together a diverse group of researchers from multiple disciplines in Limburg, Germany. On this occasion, various scientists contributed to a fruitful exchange of ideas across diverse fields focusing on biological and artificial surfaces. The symposium highlighted significant progress in the field of biomimetics and served as an interdisciplinary forum for exploring emerging trends related to biological and bioinspired surfaces. The topics covered ranged across various aspects of biomimetic research from characterization of biological functions to bioinspired technical applications, with central themes including micro- and nanostructured bioinspired surfaces and their tribological characteristics (friction, wear resistance, and adhesion), as well as self-cleaning and optical properties, underscoring the overarching aim of the symposium to integrate biological phenomena and materials science towards biomimetic approaches. The fruitful discussions and collaborations from this symposium led to the motivation of vigorously representing this area in the *Beilstein Journal of Nanotechnology*, resulting in more than just one thematic issue showcasing novel research in this field.

Emerging from this event, a first thematic issue "Biomimetics and Bioinspired Surfaces: From Nature to Theory and Applications" collected a range of studies by symposium attendees and further international colleagues on the scope of the symposium [4]. Due to the enthusiasm of the community and high number of submissions, covering a broad scope related to insect adhesion [5–7], functions of animal surfaces [2,8], and timely reviews on bioinspired nanotechnology [9–11], it was decided to continue with a second thematic issue. This second thematic issue was initiated to address the great interest across readers and authors that took note of the previous issue as a follow-up with an extended scope to continue representation of nano- and microstructure effects in biological and bioinspired systems in the *Beilstein Journal of Nanotechnology*.

The present thematic issue entitled “Micro- and Nanoscale Effects in Biological and Bioinspired Materials and Surfaces” consists of 15 articles representing all facets of research on biological and bioinspired surfaces, ranging from experimental and microscopy studies on biological objects, translational approaches with characterization of artificial replicas, bioinspired prototypes to theoretical studies including modeling and reviews of the state-of-the-art in various fields of bioinspired nanotechnologies.

A range of original research articles on biological phenomena provides a strong foundation to deepen our knowledge on functional biological systems and to open new pathways for innovative technological advances. These cover various different groups of organisms, such as animal functions in insects [12–13], polychaete worms [14], crustaceans [15], and geckos [16], and examine different functional aspects with particular technological interest including cleaning [12], adhesion [14–16], and structural coloration [13].

Piersanti et al. [12] described the functional morphology and material composition of the cleaning devices of a damselfly species and experimentally investigated their performance. This species bears specialized grooming structures on the tibiae of its forelegs with dedicated micro- and nanostructures aiding in efficient cleaning of the eyes and antennae of this insect to maintain sensory functions. Another insect study by Lopez et al. [13] investigated the ultrablack cuticle of a velvet ant using a range of microscopy methods and optical spectrometry providing structural and spectrometric information on the design of natural ultrablack materials.

Adhesion is a widespread topic in the field of nanotechnology and subject to various studies investigating the relationships between structures and functions. As the physical constraints for adhesive systems are omnipresent for animals and plants, but require sophisticated embedding into the ecological context, comparison across different organisms and their respective ecology allows for deeper understanding of the fine-tuning of micro- and nanostructures involved in adhesion [17]. Underwater adhesion is challenging and subject to chemical demands of the medium in which adhesives need to properly function. Two studies on different proteinaceous invertebrate adhesives exemplify recent advances in the field of marine bioadhesion. Duthoo et al. [14] studied the complex composite cement of a tube-dwelling annelid in regard to the ultrastructure and composition of the secreting cells and its cement product, providing a strong basis for further research on the assembly of biological and bioinspired adhesives. Sawant et al. [15] focused on the cement of a barnacle and experimentally investigated a specific key cement protein and its self-assembly under varying environmental parameters. For terrestrial animals, adhesive systems usually provide temporary adhesion and do not make use of cements. A study on the evolution of subdigital microstructures in *Cyrtodactylus* geckos by Ginal et al. [16] addressed how adhesive microstructures (setae) have independently evolved multiple times in this group in a comparative morphological framework with phylogenetically informed analyses.

Other sources for potential bioinspiration have been discussed in a review on the composite hydrogel-like mucilages of plant seeds by Kreitschitz and Gorb [18]. The mucilage envelope of seeds supports seed germination, protection from pathogens and predators, and attachment to various surfaces for dispersal. This three-dimensional polysaccharide network is capable of absorbing large amounts of water to function either as an efficient lubricant or a strong adhesive depending on its water content, and bears potential for direct industrial applications or inspiration for novel technical adhesives.

Some contributions highlighted the explanatory power of computational analysis of biological functional materials. Here, biological phenomena were investigated based on characterization of functional plant tissues [19] integrating experiments and finite element models, and animal tissues [20] incorporating molecular dynamics and finite element methods. Investigating the anisotropic hygroscopic behavior of the involved materials in bending in pine cone scales, Ulrich et al. [19] provided insights into biological plant materials combining experiments with simulations that provide inspiration for biomimetic actuators. For animals, Jain et al. [20] applied a multiscale computational model to gain detailed insights into the molecular and mechanical behavior of gecko setae during adhesion. They combined molecular dynamics for the simulation of the adhesive contact between the gecko spatula and the substrate with finite element modeling of the mechanical behavior of the adhesive seta to understand key aspects of gecko seta adhesion across scales.

Besides these studies, focusing on sources for bioinspiration based on the understanding of micro- and nanoscale effects in biological systems (“biology push”), numerous advances were achieved on replication of biological principles into artificial replicas [21], as well as studies on bioinspired materials and fabrication methods [22–23]. For instance, Farran et al. [21] characterized orientation-dependent photonic bandgaps in scales of gold-dust weevils and titania-based artificial mimics of these scales obtained from sol–gel replication, aiding in the production of customizable photonic bandgaps for novel optical materials.

On the application-driven side (“technology pull”) of bioinspired engineering, two studies demonstrated approaches based on technical advances informed by biological systems. Material structure and properties of biological systems are often considered a source of inspiration for construction of durable engineered materials. In this context, González et al. [22] investigated functionally graded polypropylene-based materials and metamaterials inspired by the interfacial connection between the hemiparasitic mistletoe and its plant host. Their glass-fiber-reinforced polypropylene mimics of the mistletoe–host connection were mechanically characterized, and the derived principles were transferred to metamaterials that present promising advances for programmable local strain and failure behavior. A strength of such a technology-pull approach for problem solving is that principles extracted from different biological templates can be combined to benefit from the effects observed in both systems. This was demonstrated in the biomimetic approach of Faase et al. [23] which utilized slippery liquid-infused porous surfaces inspired by carnivorous pitcher plants with polydopamine anchorage inspired by mussel adhesive systems to produce surface passivation materials that avoid clot formation in a biomedical context.

Lastly, the issue includes reviews on applications of such bioinspired nanotechnologies for cultural heritage protection [24], medical therapeutic treatments [25–26], and vitamin B12 biosensing [27]. Lama et al. [24] presented a thorough review on the most recent scientific literature on techniques, materials, and processes used in the production of coatings for cultural heritage protection with particular attention to the generation of superhydrophobic surfaces. The challenges for cultural heritage protection are two-sided, as not only efficient repellency is required, but also the protected object needs to be conserved without risk of damage caused by the treatment. Other reviews focused on various nanotechnological applications in medicine and pharmaceutics. Highlighting advances in biomimetic medicine, Perini et al. [25] reviewed the importance of biomimetic nanocarriers for drug delivery systems with improved biocompatibility and target specificity, whereas Mohammed et al. [26] focused on biomimetic potential for nanomedicines in tumor therapy. Besides nanotechnological solutions inspired by biological surface features to improve transport and effects of drugs, biological systems can be suitable templates for recognition of biomolecules. The review by Gharibzahedi and Altintas [27] provides a timely and comprehensive overview of recent advances in optical biosensing technologies for the detection of vitamin B12. The reviewed approaches, with a particular focus on bioinspired and nanomaterial-based platforms, address the needs for fast, reliable, and noninvasive detection of specific nutrients in food industry and pharmaceutics.

This thematic issue offers a distinctive set of new concepts and data on biological and bioinspired surfaces, creating a strong link between biological research and nanotechnology. The high-quality contributions highlight cutting-edge designs as well as specific technological implementations of biomimetic surface structures. Our gratitude goes to all authors for sharing their work using this paper collection and to the reviewers for their critical input, which substantially strengthened the quality of the manuscripts. We hope that the articles in this collection motivate researchers and developers to push biomimetic design further, opening new angles in materials research and technology development.

Thies H. Büscher, Rhainer Guillermo Ferreira, Manuela Rebora, and Stanislav N. Gorb

Kiel, Uberaba, and Perugia, January 2026

## Data Availability

Data sharing is not applicable as no new data was generated or analyzed in this study.
